# Supplementary material for: The Role of the Tyrosine-Based Sorting Signals of the ORF3a Protein of SARS-CoV-2 on Intracellular Trafficking, Autophagy, and Apoptosis
Source: bioRxiv. 2023 Jul 24:2023.07.24.550379. Preprint. [Version 1] doi: 10.1101/2023.07.24.550379 (PMC10402054; doi:10.1101/2023.07.24.550379)
Supplement: Supplement 1 [file NIHPP2023.07.24.550379v1-supplement-1.pdf]

bioRxiv preprint doi: https://doi.org/10.1101/2023.07.24.550379; this version posted July 24, 2023. The copyright holder for this preprint (which was not certified by peer review) is the author/funder, who has granted bioRxiv a license to display the preprint in perpetuity. It is made available under aCC-BY 4.0 International license.

```
            151     160                                   200       212
             |       |                                     |         |
Tor2        THNYDYCIPYNSVTDTIVVTEGDGISTPKLKEDYQIGGYSEDRHSGVKDYVVVHGYFTEVYYQLESTQIT
Civet007    THNYDYCIPYNSVTDTIVVTEGDGISTPKLKEDYQIGGYSEDRHSGVKDYVVVHGYFTEVYYQLESTQIT
WIV1        THNYDYCIPYNSVTDTIVVTAGDGISTPKLKEDYQIGGYSENWHSGVKDYVVVHGYFTEVYYQLESTQIT
WIV16       THNYDYCIPYNSVTDTIVVTAGDGISTPKLKEDYQIGGYSENWHSGVKDYVVVHGYFTEVYYQLESTQIT
Rs3367      THNYDYCIPYNSVTDTIVVTAGDGISTPKLKEDYQIGGYSENWHSGVKDYVVVHGYFTEVYYQLESTQIT
RsYN09      TNCYDYCIPYNSVTDTIVLTSSDGTNVPKLKEDYQIGGYSEDWHSGVKDYVVIHGYFTEIYYQLESTQLS
RsYN03      TNCYDYCIPYNSVTDTIVLTSSDGTNVPKLKEDYQIGGYSEDWHSGVKDYVVIHGYFTEIYYQLESTQLS
Rs7327      THNYDYCIPYNSVTDTIVVTAGDGISTPKLKEDYQIGGYSENWHSGVKDYVVVHGYFTEVYYQLESTQIT
Rs4874      THNYDYCIPYNSVTDTIVVTAGDGISTPKLKEDYQIGGYSENWHSGVKDYVVVHGYFTEVYYQLESTQIT
Rm1/2004    TNCFDYCIPYNSITDTIVLTSGDGTTQPKLKEDYQIGGYSEDWHSGVKDYVVIHGYFTEIYYQLESTQLS
JTMC15      TYNYDYCIPYNSVTDTIVVTTGDGISTPELKEYYQIGGYSEDWHSGVKDYVVVHGYFAEVHYQLESTQIT
JL2012      TYNYDYCIPYNSVTDTIVVTTGDGISTPELKEYYQIGGYSEDWHSGVKDYVVVHGYFAEVHYQLESTQIT
Rf1/2004    TYNYDYCIPYNSVTDTIVVTSGDGISTPELKEDYQIGGYSEDWHSGVKDYVVVHGYFTEVHYQLESTQIT
BtSY1       THNYDYCLPYNSVTETIVVTAGDGISTPKLKEDYQIGGYSEDWHSGVKDYVVIHGYFTEVYYQLESTQIT
HKU3-13     TNNYDYCIPYNSVTDTVVITSGDGTNQPKLKEDYQIGGYSEDWHSGVKDYVVIYGYFTEVYYQLESTQLS
As6526      THNYDYCIPYNSVTDTIVVTAGDGISTPKLKEDYQIGGYSEDWHSGVKDYVVVHGYFTEVYYQLESTQIT
LYRa11      TNCYDYCIPYNSVTDTIVLTSSDGTNVPKLKEDYQIGGYSEDWHSGVKDYVVIHGYFTEIYYQLESTQLS
Rs9401      THNYDYCIPYNSVTDTIVVTAGDGISTPKLKEDYQIGGYSENWHSGVKDYVVVHGYFTEVYYQLESTQIT
```

Supplemental Figure 1. Eighteen ORF3a sequences from SARS-CoV (strain Tor2), Civet (Civet007) and SARS-CoV-like strains (all 274 amino acids in length). Shown are amino acids 160 to 240 with the potential tyrosine-based sorting motifs (in red). The species from which the isolate was obtained and accession numbers are: Tor2 (*Homo sapiens*; YP_009825052); Civet007 (*Paradoxurus hermaphroditus*; AAU04635); WIV1 (*Rhinolophus sinicus*; AGZ48832); WIV16 (*Rhinolophus sinicus*; ALK02458); Rs3367 (*Rhinolophus sinicus*; AGZ48819); RsYN09 (Rhinolophus stheno; QWN56264); RsYN03 (Rhinolophus sinicus; QWN56233); Rs7327 (*Rhinolophus sinicus;* ATO98219); Rs4874 (*Rhinolophus* sinicus; ATO98206); Rm1/2004 (*Rhinolophus macrotis*; ABD75326); JTMC15 (Rhinolophus ferrumequinum; ANA96028); JL2012 (*Rhinolophus ferrumequinum*;AIA62278); Rfl/2004 (*Rhinolophus ferrumequinum*; ABD75316); BtSY1 (*Rhinolophus thomasi;* WBV74274); HKU3-13 (*Rhinolophus spp*. ADE34824); As6526 (*Aselliscus stoliczkanus;* ATO98109); LYRa11 (Rhinolophus affinis; AHX37559); Rs9401 (*Rhinolophus sinicus;* ATO98232).
